# Voice phenomenology as a mirror of the past

**DOI:** 10.1017/S0033291721004955

**Published:** 2023-05

**Authors:** David van den Berg, Eva Tolmeijer, Alyssa Jongeneel, Anton B. P. Staring, Eline Palstra, Mark van der Gaag, Amy Hardy

**Affiliations:** 1 Department of Clinical Psychology, VU University and Amsterdam Public Health Research, van der Boechorststraat 7, 1081 BT Amsterdam, The Netherlands; 2 Department of Psychosis Research, Parnassia Psychiatric Institute, Zoutkeetsingel 40, 2512 HN The Hague, The Netherlands; 3 ABC Department for First Episode Psychosis, Altrecht Psychiatric Institute, ABC straat 8, 3512 PX Utrecht, The Netherlands; 4 Department of Psychology, Institute of Psychiatry, Psychology & Neuroscience, King's College London, De Crespigny Park, London SE5 8AF, UK; 5 South London & Maudsley NHS Foundation Trust, Bethlem Royal Hospital, Monks Orchard Road, Beckenham, Kent BR3 3BX, UK

**Keywords:** Voices, auditory hallucinations, trauma, victimisation, phenomenology, psychosis, schizophrenia

## Abstract

**Background:**

Post-traumatic mechanisms are theorised to contribute to voice-hearing in people with psychosis and a history of trauma. Phenomenological links between trauma and voices support this hypothesis, as they suggest post-traumatic processes contribute to the content of, and relationships with, voices. However, research has included small samples and lacked theory-based comprehensive assessments.

**Method:**

In people with distressing voices (*n* = 73) who experienced trauma prior to voice-hearing, trauma–voice links were assessed both independently and dependently (descriptions were presented and rated separately and together, respectively) by both participants and researchers. A structured coding frame assessed four types of independent links (i.e. victimisation type, physiological-behavioural, emotional, and cognitive response themes including negative self-beliefs) and three types of dependent links: relational (similar interaction with/response to, voice and trauma); content (voice and trauma content are exactly the same); and identity (voice identity is the same as perpetrator).

**Results:**

Independent links were prevalent in participants (51–58%) and low to moderately present in researcher ratings (8–41%) for significant themes. Identification of negative self-beliefs in trauma was associated with a significantly higher likelihood of negative self-beliefs in voices [participants odds ratio (OR) 9.8; researchers OR 4.9]. Participants and researchers also reported many dependent links (80%, 66%, respectively), most frequently relational links (75%, 64%), followed by content (60%, 25%) and identity links (51%, 22%).

**Conclusion:**

Trauma appears to be a strong shaping force for voice content and its psychological impact. The most common trauma–voice links involved the experience of cognitive-affective psychological threat, embodied in relational experiences. Trauma-induced mechanisms may be important intervention targets.

## Introduction

‘My parents used to emotionally and physically abuse me. My father often insulted me. He would say: ‘You're a schmuck’. He beat me and instructed my mother to beat me up. I thought: ‘Why is this happening? I hope they go away and never come back’. I felt scared, sad, and helpless. I also felt my stomach contracting. […] Today, I hear my father's voice insulting me and commenting in a negative way: ‘You're a schmuck’, ‘You're an asshole’, and ‘You're not doing it right’. I then think ‘Go away’. I feel inferior and get startled. Physically I feel tense’. (Quote from a voice-hearing participant).

The relationship between trauma and psychosis is now well recognised, and evidence suggests there may be specific associations between interpersonal victimisation and voice hearing (Hardy, 2017; Morrison, Frame, & Larkin, 2003; Van Den Berg et al., 2015; Varese et al., 2012). Trauma-focused cognitive behavioural models postulate that post-traumatic psychological mechanisms give rise to hallucinatory experiences, which suggests there should be phenomenological similarities between the experience of traumatic events and voice-hearing (e.g. Garety, Kuipers, Fowler, Freeman, & Bebbington, 2001; Hardy, 2017; Morrison *et al*. 2003).

Phenomenological links between traumatic events and hallucinations have been studied several times (Corstens & Longden, 2013; Hamner, 1997; Hardy et al., 2005; Jessop, Scott, & Nurcombe, 2008; Mueser & Butler, 1987; Peach et al., 2020; Raune, Bebbington, Dunn, & Kuipers, 2006; Read & Argyle, 1999; Scott, Nurcombe, Sheridan, & McFarland, 2007). In the two most methodologically robust studies (Hardy et al., 2005; Peach et al., 2020), the authors developed coding frames to systematically investigate links between the phenomenology of trauma and hallucinations. In both studies, ratings were completed by two independent raters to allow assessment of inter-rater reliability. Operationalised definitions assessed two possible links: direct or content links hypothesised to relate primarily to episodic memory intrusions (e.g. emotional abuse involving comments ‘you are ugly’ and voices saying ‘you are ugly’), and indirect or thematic links hypothesised to be driven by schematic beliefs and emotional regulation (e.g. witnessing and fearing injury and voices threatening to hurt the person). Hardy et al. (2005) found that in people who reported past trauma that was currently affecting them, researchers rated 13% of hallucinations as having content directly related to the trauma and 45% of hallucinations as thematically related to trauma. People with voices reflecting an intrusive and controlling theme were more likely to have experienced traumas with a similar theme. Higher rates of phenomenological links were identified by Peach et al. (2020), who found that 33% experienced hallucinations with content directly related to their traumas and 67% reporting hallucinations with content thematically related to their traumas. These higher rates are likely attributable to the fact that multiple traumatic events and hallucinations were coded for links in the latter study.

Corstens and Longden (2013) identified phenomenological links by asking voice-hearers to report on the identity of their voices and of their trauma perpetrators. They found that 45% of participants attributed the identity of their voice to an abusive family member and 23% to other perpetrators. However, other types of phenomenological links beyond identity were not examined. Other studies investigated phenomenological links using structured interviews (Hamner, 1997; Mueser & Butler, 1987; Scott et al., 2007), clinician-administered questionnaires (Jessop et al., 2008), and chart review (Read & Argyle, 1999). These studies used varied definitions of phenomenological links including: correspondence between the identity of the hallucination and the perpetrators (Scott et al., 2007), thematic correspondence (Jessop et al., 2008; Read & Argyle, 1999), and exact content overlap (Hamner, 1997; Mueser & Butler, 1987).

It is evident that despite the growing body of research exploring phenomenological links between trauma and hallucinations, studies used varied definitions of these links, and different measures of assessment, employing either voice-hearer or researcher ratings. Samples have also tended to be small [*n* = 40 (Hardy et al., 2005); *n* = 36 (Peach et al., 2020)] and most studies were conducted post hoc. To enhance methodological robustness, this study used a structured way of capturing trauma and voice-hearing experiences to assess theory-based trauma–voice links, from both voice-hearer and researcher perspectives.

In this study, we developed a theory-driven coding frame and used this to elicit structured descriptions of trauma and voice-hearing experiences and comprehensively assess phenomenological links between trauma and voice-hearing, based on cognitive-behavioural models of post-traumatic stress in psychosis (e.g. Garety *et al*. 2001; Hardy, 2017; Morrison *et al*. 2003). This study aimed to examine the occurrence of two types of links assessed by the coding frame: (1) independent links: i.e. between victimisation type, physiological-behavioural response, emotional response, and cognitive response themes of traumas and voices; and (2) dependently perceived links: i.e. relational links (i.e. similar interaction with, and response to, the voice and trauma), identity links (voice identity is the same as the perpetrator of the trauma), and content links (voice content is exactly the same as the trauma).

## Method

### Participants

Participants were recruited from outpatient mental healthcare teams for people with long-term and complex mental health difficulties. They were recruited as part of the Temstem trial [a multicentre randomised controlled trial testing the effects of a smartphone application (Temstem) for people with persistent, frequent and distressing voices, ISRCTN 75717636, Jongeneel et al., 2018b]. This study was approved by the medical Ethics Committee of the VU University Medical Centre (METC number: 2015.435/NL53684.029.15). Inclusion criteria for the current study were: (1) adults who experience voices ⩾1 month for at least 4 days a week in at least three of the last 4 weeks, and (2) experience of the most distressing interpersonal trauma before the onset of voices.

**Fig. 1.**
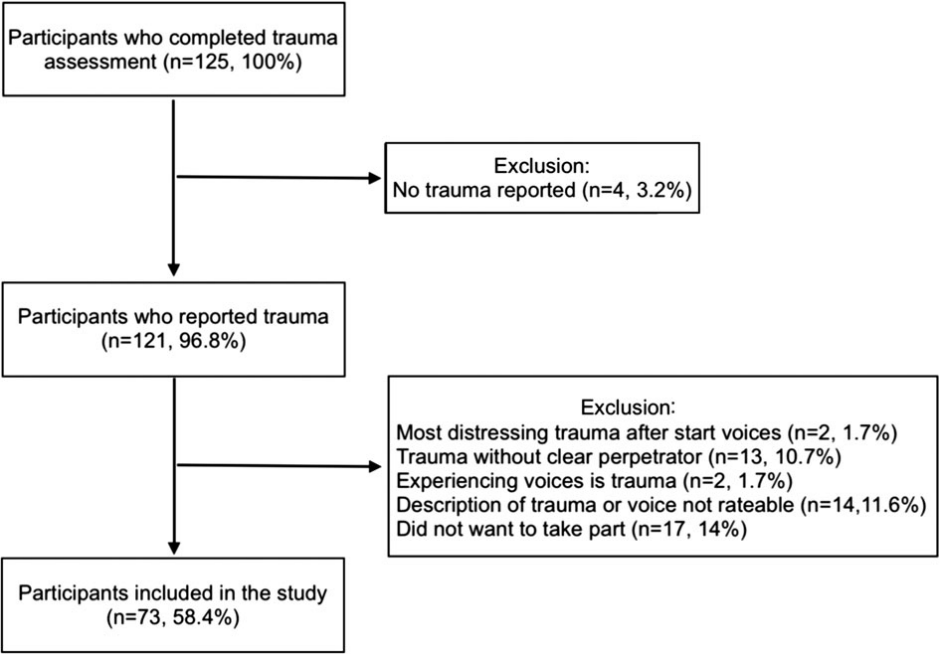
Participant flowchart.

The participant flowchart is presented in Fig. 1. Nearly all 125 participants (97%) experienced traumatic event(s), and for most (84%) these included interpersonal events. Seventy-three participants (58%) experienced their most distressing interpersonal traumatic event before the onset of voices and were willing and able to provide a description of this trauma and their most distressing voices. Most participants were female (62%). Mean age was 42.5, s.d. = 11.9. The majority of participants were born and had parents who were born in The Netherlands (62%), followed by participants who were born or had at least one parent born in Africa (12%), South America (8%), Europe (7%), Asia (6%), Caribbean Islands (3%), or other countries (1%). The majority of participants completed pre-vocational education (25%) or secondary vocational education (27%). Participants most frequently had a primary diagnosis of schizophrenia (39%), psychotic disorder not otherwise specified (25%), or schizoaffective disorder (12%). The majority of participants had not received cognitive behaviour therapy (64%).

### Measures

*The Trauma and Life Events* (*TALE*) *checklist* (Carr, Hardy, & Fornells-Ambrojo, 2018) was developed for people with psychosis and assesses 20 types of traumatic and adverse life events [war trauma, attachment disrupting events (e.g. loss of a parent due to death or being placed in care), bullying, discrimination, physical abuse, emotional abuse, neglect, sexual abuse, psychosis-related traumas (e.g. the use of force during a hospital admission and experiencing or witnessing threatening or shocking events while in care), and accidents]. Participants report whether they experienced an event (‘Yes’ or ‘No’ response), whether this occurred repeatedly (‘Did it happen more than once?’), and the approximate age or age range at time of the event(s).

*The Psychotic Symptom Ratings Scales* (Haddock, McCarron, Tarrier, & Faragher, 1999; Woodward et al., 2014). The voices subscale was used which has 11 items, rated on a 5-point Likert scale. These items measure voice characteristics including dimensions of distress (distress, negative content, and control), frequency (frequency, duration, and disruption), attribution (location and origin of voices), and loudness.

*The Structured Coding Frame* was developed by the authors of this study, to comprehensively assess phenomenological links between interpersonal traumatic events and voice hearing (see online Supplementary materials). Items were generated by the authors reviewing existing models and empirical evidence. The first section consists of 18-items and assesses the victimisation type, physiological-behavioural response, emotional response, and cognitive response themes associated with voice-hearing and interpersonal traumatic events (scored for both separately). Each item is rated on a scale of 0 (‘No, not at All’) to 5 (‘Yes, very much’). These ratings can then be coded for the presence or absence of an independent correspondence between each trauma and voice theme. The second section directly assesses dependent perceptions of phenomenological links between trauma and voices. This includes 5-items on identity links (i.e. *Do the voices sound like or belong to people involved in any of the events?*), content links (i.e. *Do the voices ever say exactly the same things that were said to you during the events?*) and relational links between trauma and voices (i.e. *Is the way the voices treat you or the way that they behave similar to the way people treated you during any of the events?*; *Do you react to or cope with the voices in a similar way to how you reacted or coped during any of the events?*; *Do the voices make you feel the same as how you felt during the events?*), scored on a scale from 0 ‘No, not at all’ to 5 ‘Yes, very much’.

### Procedure

#### Voice-hearer assessment of trauma and voice phenomenology

All participants were administered the PSYRATS voices subscale as part of the baseline assessment. The subsequent assessment of trauma and voice phenomenology by participants followed six steps. First, participants formulated a description of their main distressing voice(s) using 100 words maximum. This description was formulated by asking participants to identify their main distressing voice(s) and prompting them to provide a structured description in relation to (1) identity; (2) content; (3) appraisals; (4) emotional responses; (5) physiological responses; and (6) behavioural responses. Second, participants completed questions in relation to this description of their voice(s) experience using the first independent part of the coding frame about voices (see online Supplementary materials). Third, participants then completed the TALE and identified their most distressing interpersonal trauma before the onset of voices. Fourth, they were again prompted to provide a structured description using 100 words maximum, this time of their identified trauma, in relation to (1) perpetrator identity; (2) content; (3) appraisals; (4) emotional responses; (5) physiological responses; and (6) behavioural responses. Fifth, they then completed the first independent part of the coding frame in relation to their experience of this traumatic event. Sixth, participants then completed the second dependent part of the coding frame to assess their personal views on identity, relational, and content links between their most distressing trauma and voice experiences.

#### Researcher assessment of trauma–voice phenomenology

The assessment of trauma and voice phenomenology by the researchers followed three steps. First, the trauma and voice descriptions were rated independently by two researchers (TS and AH) using the first part of the coding frame adapted for researchers (see online Supplementary materials) which assesses the phenomenology of voices and trauma, respectively. Basic rating guidelines were formulated prior to rating of the frames. Guidelines were refined during consensus rating. The final rating guidelines for the independent links are presented in Table 1. To ensure independent assessment, trauma and voice descriptions were presented separately and randomly so it was not possible to ascertain which traumas and voices were associated (belonged to the same individual). Second, ratings were compared, any disagreements were resolved by consensus, and themes were scored as either present ‘yes’ or absent ‘no’, in discussion with DvdB and AJ. Third, the raters were then provided with the paired descriptions of both the interpersonal traumas and voices and dependently rated the items in the second section of the coding frame adapted for researchers examining content, identity, and relational links. For the dependent links the following rating guidelines applied: (1) relational links were scored if there was a similar interaction with or response to voice(s) and trauma, (2) content links were scored if the voice content was exactly the same as the trauma content, and (3) identity links were scored if the voice identity was the same as the perpetrator identity.

**Table 1.** Definitions for independent links

Independent links	Definition
Victimisation type	
Criticism/blame	Criticism or blame, including negative comments.
Humiliation	Being socially devalued in the presence of others.
Violence	Actual or threatened violence.
Coercion	Coercion including being involuntarily confined.
Physiological-behavioural response	
Freeze	Emotional detachment, feeling numb, freezing, depersonalisation, derealisation, fainting, or tonic immobility.
Ignoring	Coping by ignoring.
Flight	Running/fleeing/avoiding/negative experiential avoidance.
Submission	Passive response.
Fight	Fighting, resisting, or attempting to control the situation. Thinking out loud that the voices were wrong was also considered to be resistance if clearly directed to the voices.
Emotional response	
Anger, anxiety, sadness, shame, guilt	Clear reference to the emotion (anger, anxiety, sadness, shame, or guilt).
Cognitive response	
Powerless, threatened, fear of rejection, negative self	Clear reference to the cognition, or the emotion and/or behaviour strongly implies an associated cognitive response.

### Analyses

Given the exploratory nature of the study, all links were examined using descriptive statistics. Independent links between trauma and voice phenomenology (e.g. including victimisation type, physiological-behavioural response, emotional response, and cognitive response themes) were analysed using the first section of the coding frame assessing the phenomenology of voices and trauma when descriptions were presented separately. Dependent phenomenological links (i.e. relational, content, and identity links) were analysed using the second part of the coding frame assessing the phenomenology of voices and trauma when descriptions were presented together. To support coding of the links, the Likert scores of participants were dichotomised to 0–1 ‘No’ and 2–5 ‘Yes’ based on the distribution of the data and reasoning that reporting ‘1’ out of 5 is not sufficiently indicative of presence. Odds ratio (OR) estimates were calculated to investigate the odds of endorsing a theme in the voice phenomenology given endorsement of this same theme in the trauma phenomenology. These analyses were only carried out for themes with data that met statistical assumptions.

## Results

### Independent participant-rated phenomenological links

Virtually all participants reported traumatic experiences that preceded the onset of voices (Fig. 1). In the participant ratings, there was a high rate of independent phenomenological links between the most distressing interpersonal traumas and voices (51–95%; Table 2), with the highest rates for the themes of criticism/blame, anger/anxiety/sadness, and powerless. The data were not all suited for statistical analyses considering that some themes for participants (i.e. criticism/blame, freeze, anger, sadness, guilt, powerless, threatened, and fear of rejection) had cell values below 5. OR analyses involving the themes that met statistical assumptions (i.e. humiliation, violence, coercion, ignoring, flight, submission, fight, anxiety, shame, and negative self-beliefs) revealed that endorsement of the themes of fight, flight, and negative self-beliefs in the trauma phenomenology, were associated with a significantly higher likelihood of endorsement of fight [OR 4.7, 95% confidence interval (CI) 1.4–15.2, *p* < 0.01], flight (OR 3.8, 95% CI 1.3–11.1, *p* < 0.05), and negative self-beliefs (OR 9.8, 95% CI 3.1–30.9, *p* < 0.01) in the voice phenomenology. These significant themes were also associated with high rates of independent links (51–58%; Table 2).

**Table 2.** Independent links between themes of trauma and voices

	Participant ratings of trauma and voice experiences (*n* = 73)							Researcher ratings of trauma and voice descriptions (*n* = 73)						
	Present in voice		Present in trauma		Links between themes			Present in voice		Present in trauma		Links between themes		
Items	Freq.	%	Freq.	%	Freq.	%	OR	Freq.	%	Freq.	%	Freq.	%	OR
Victimisation type														
Criticism/blame	69	94.5	71	97.3	69	94.5	n/a	58	79.5	28	38.4	24	32.9	n/a
Humiliation	57	78.1	50	68.5	41	56.2	2.0	0	0	14	19.2	0	0	n/a
Violence	47	64.4	57	78.1	38	52.1	1.6	35	47.9	55	75.3	26	35.6	0.9
Coercion	56	76.7	40	54.8	33	45.2	1.1	44	60.3	32	43.8	21	28.2	1.5
Physiological-behavioural response														
Freeze	45	61.6	60	82.2	44	60.3	n/a	6	8.2	24	32.9	2	2.7	n/a
Ignoring	63	86.3	47	64.4	42	57.5	2.0	40	54.8	4	5.5	3	4.1	n/a
Flight	**53**	**72.6**	**49**	**67.1**	**40**	**54.8**	3.8*	17	23.3	23	31.5	2	2.7	n/a
Submission	49	67.1	53	72.6	38	52.1	2.1	**21**	**28.8**	**35**	**47.9**	**6**	**8.2**	0.3*
Fight	**56**	**76.7**	**42**	**57.5**	**37**	**50.7**	4.7**	38	52.1	26	35.6	14	19.2	1.1
Emotional response														
Anger	65	89.0	62	84.9	56	76.7	n/a	**41**	**56.2**	**42**	**57.5**	**30**	**41.1**	4.5**
Anxiety	63	86.3	63	86.3	56	76.7	3.4	39	53.4	52	71.2	29	39.7	1.4
Sadness	63	86.3	64	87.7	56	76.7	n/a	42	57.5	39	53.4	24	32.9	1.4
Shame	60	82.2	58	79.5	50	68.5	3.1	9	12.3	14	19.2	3	4.1	n/a
Guilt	63	86.3	52	71.2	50	68.5	n/a	5	6.8	7	9.6	1	1.4	n/a
Cognitive response														
Powerless	59	80.8	68	93.2	57	78.1	n/a	32	43.8	45	61.6	20	27.4	n/a
Threatened	58	79.5	68	93.2	54	74.0	n/a	16	21.9	37	50.7	8	11.0	1.0
Fear of rejection	59	80.8	53	72.6	49	67.1	n/a	1	1.4	0	0	0	0	n/a
Negative self	**50**	**68.5**	**50**	**68.5**	**42**	**57.5**	9.8**	**15**	**20.5**	**13**	**17.8**	**6**	**8.2**	4.9*

*p < 0.05, **p < 0.01. Bold lines represent statistically significant OR.

### Independent researcher-rated phenomenological links

In the researcher ratings of the provided trauma and voice descriptions, there was a low to moderate rate of independent links between trauma and voice phenomenology (0–41%; Table 2), with the highest rates for the themes of violence, criticism/blame, anxiety, sadness, anger, and powerless. Also, in the researcher ratings, the data of several themes (i.e. criticism/blame, humiliation, freeze, ignoring, flight, shame, guilt, powerless, and fear of rejection) were not suited for statistical analyses due to cell values below 5. OR analyses involving the themes that met statistical assumptions (i.e. violence, submission, fight, anger, anxiety, sadness, threatened, and negative self-beliefs) revealed that endorsement of the themes of anger and negative self-beliefs in the trauma phenomenology, was associated with a significantly higher likelihood of endorsement of anger (OR 4.5, 95% CI 1.7–12.3, *p* < 0.01) and negative self-beliefs (OR 4.9, 95% CI 1.3–17.8, *p* < 0.05) in the voice phenomenology. Contrarily, endorsement of the theme of submission was associated with a significantly lower likelihood of endorsement of submission in the voice phenomenology (OR 0.32, 95% CI 0.11–0.95, *p* < 0.05). These significant themes were associated with moderate rates of independent links (8–41%; Table 2).

### Dependent phenomenological links

In the dependent section of the coding frame assessment, i.e. when trauma and voice experiences were presented together, both participants and researchers frequently identified at least one type of link between traumatic events and voices. Relational links were most commonly reported by both participants and researchers, subsequently followed by content and identity links (see Table 3 for examples of links and Table 4 for prevalence rates).

**Table 3.** Examples of trauma and voices descriptions and phenomenological link(s)

Relational link	
Most distressing trauma	Most dominant or distressing voice
*I have been emotionally abused by my father. He would also make sexual comments about me or my female friends: ‘I want to have sex with them’. I thought ‘men only want sex’. I felt hopeless, ashamed, and angry. Due to my past, I have developed severe problems in my intimate relationships.*	*I hear the voice of a stalker. He insults and threatens me. He keeps wanting to have sex with me: ‘there isn't a day that goes by where I don't think of you’. I then think: ‘oh, it's him again’ ‘I am weak’. He makes me feel anxious, scared, and sad. When I hear him, I experience panic attacks and pain. I want to become stronger by going to the gym to fight him.*
Relational and identity link	
Most distressing trauma	Most dominant or distressing voice
*My father abused me physically and sexually on a daily basis. I have felt frightened and unsafe during my entire childhood because of the abuse. When my father abused me, I dissociated from my body and watched myself from above. I often made sure that my brother slept next to me, and I avoided my father.*	*I hear the voices of my father and brother. The voices insult me and comment on what I am doing or how I look. They also command me to do or not do things. I feel powerless, guilty, and used. It consumes my energy. Sometimes I also get panic attacks where it feels like I am leaving my body. Nevertheless, I try to do what I want to do and ignore the voices as much as possible.*
Relational and content link	
Most distressing trauma	Most dominant or distressing voice
*During my entire time at school, I've been bullied by several schoolmates. It was both emotional and physical abuse. They punched and kicked me and spat on me. They told me ‘You are ugly and fat’. I thought: ‘Why are they doing this?’. I felt lonely and misunderstood. I was angry and disappointed. I ignored it and let it happen.*	*I hear a voice with the sound of my mother. The voice insults me and gives me assignments and comments on me. The voice says: ‘You are fat’ and ‘Grab that knife’. I then think: ‘Why are you doing this, why are you bothering me?’. I then feel angry and sad. I start to shiver and tremble. Afterwards I meditate or lie on my bed with my eyes closed to calm down.*
Relational, identity, and content link	
Most distressing trauma	Most dominant or distressing voice
*My parents emotionally and physically abused me. My father insulted me: ‘You're a schmuck’. He beat me and instructed my mother to beat me up. I thought: ‘Why is this happening?’ ‘I hope they go away and never come back’. I felt scared, sad, and helpless. I also felt my stomach contracting. I let it happen. Afterwards I was sent to my room where I laid down in bed.*	*I hear my father's voice which insults me and comments on me negatively. He says things such as: ‘You're a schmuck’, ‘You're an asshole’ and ‘You're not doing it right’. I then think: ‘Go away’. I feel inferior and get startled. Psychically I feel tense. I immediately react by swearing with the word: ‘Judaism’.*

**Table 4.** Dependent links between trauma and voices

	Participant rating of trauma and voice experiences		Researcher rating of trauma and voice descriptions	
Links	Freq.	%	Freq.	%
Any	58	79.5	48	65.8
Relational	55	75.3	47	64.4
Content	44	60.3	18	24.7
Identity	37	50.7	16	21.9
Relational and content and identity	31	42.5	8	11.0

## Discussion

This study set out to investigate the phenomenology of trauma and voices in a group of people with distressing voices. The majority (95%) experienced trauma before the onset of voice-hearing. Links between interpersonal trauma and voices were commonly observed by both participants and researchers, even when links were independently coded from separate ratings of trauma and voice phenomenology. The sample was larger than included in previous coding frame studies, and trauma–voice links were assessed more comprehensively than in prior research (Corstens & Longden, 2013; Hamner, 1997; Hardy et al., 2005; Jessop et al., 2008; Mueser & Butler, 1987; Peach et al., 2020; Raune et al., 2006; Read & Argyle, 1999; Scott et al., 2007). Namely, the descriptions of trauma and voices provided to researchers were formulated *a priori* and included at least identity, content, appraisals, and emotional and physiological-behavioural responses. Additionally, both participants and researchers rated both independent and dependent phenomenological links between trauma and voices using a structured theory-driven coding frame.

The findings reveal that participants and researchers reported phenomenological links to a similar extent, although participants had higher numbers of phenomenological links, possibly as they were aware of the entirety of their experiences – their cumulative history of traumatic and voice-hearing experiences and associated thoughts, emotions, and meaning – beyond the brief descriptions provided for researchers. Alternatively, it may be that our inherent tendency to attribute our intrapsychic experiences to psychosocial causes led voice-hearers to overestimate the phenomenological links. For the independent links, both participants and researchers more commonly reported links between victimisation types (i.e. criticism/blame), emotional response themes (i.e. fear/anger/sadness), and cognitive response themes (i.e. powerless) than links with physiological-behavioural response themes. For the dependent links, both participants and researchers most commonly reported relational links, followed by content and identity links, which is in line with previous research (Hardy et al., 2005; Peach et al., 2020).

The present methodology does not allow for revealing a causal relationship between the experience of trauma and voice-hearing. However, the findings do demonstrate that people who hear voices frequently perceive at least some type of link between their voices and previous trauma, with relational links being the most common dependent link (e.g. experiencing blame in various ways), and with 50% of voices having an identity linked to a trauma perpetrator. Caveated by inherent overlap between independently rated themes and many themes being either highly prevalent or absent, the presence of the emotional, physiological-behavioural, and cognitive response themes in the trauma phenomenology – including anger, fight, flight, and negative self-beliefs, were associated with significantly higher likelihood of being present in the voice phenomenology. Previous research has found that trauma-related psychological mechanisms including posttraumatic avoidance and hyperarousal (Hardy et al., 2016) and negative self-beliefs (Hardy, O'Driscoll, Steel, Van Der Gaag, & Van Den Berg, 2020; Peach, Alvarez-Jimenez, Cropper, Sun, & Bendall, 2019) mediate the link between trauma and hallucinations. Additionally, the evidence base, including prospective studies (Daalman et al., 2012; Radua et al., 2018; Varese et al., 2012) and studies revealing a dose–response relationship between trauma and voice-hearing (Alsawy, Wood, Taylor, & Morrison, 2015; Croft et al., 2019) are considered indicators of a causal connection. Together with research revealing heterogeneity within voice-hearers who experienced trauma (Hardy *et al*. 2005; Peach *et al*. 2020), the finding that perceived links between trauma and voices are common does provide some support for a causal relationship in at least a subgroup of voice hearers.

The prevalence of types of independent links suggests that the phenomenology of trauma and voices is predominantly characterised by cognitive-affective psychological threat that becomes embodied in the relational experience. This is supported by research that the onset of voices often occurs in a traumatic context and that voices are most commonly abusive/violent and associated with feelings of anxiety and fear (Alderson-Day et al., 2020). Cognitive-affective themes encoded in autobiographical memory appear to be replayed or enacted in the voice-hearing experience which impacts on the content and relational context of voices (Brewin, Gregory, Lipton, & Burgess, 2010). Vivid feelings of threat encoded in trauma memory may lead to negative judgements about self, others, and the world which shape the content of voices by mirroring interactions with others in the past (Hardy, Van De Giessen, & Van Den Berg, 2019; Jongeneel, Pot-Kolder, Counotte, van der Gaag, & Veling, 2018a; McCarthy-Jones et al., 2014). Accordingly, trauma-related beliefs have been found to mediate the link between trauma, post-traumatic stress disorder, and hallucinations (Hardy et al., 2020; Peach et al., 2019) and the effects of trauma-focused therapy for people with psychosis (van der Vleugel et al., 2020).

Inversely, voices with greater similarity to the trauma may also activate memories about being threatened by others, which in turn could impact on how voices are appraised and responded to (Hardy, 2017; 2019). Habitual emotional regulation strategies and cognitive-behavioural responses that developed as understandable attempts to survive past traumatic experiences may be triggered 2017. In addition to the aforementioned pathways hypothesised to underlie the independent trauma and voice links, it is possible that some content links arise from de-contextualised reexperiencing symptoms such that the person hears exactly what the perpetrator said (Hardy et al., 2019; Steel, Fowler, & Holmes, 2005). When the contextual level of the trauma memory encoding is strongly disrupted, the information at the sensory-perceptual information may intrude without awareness of recollecting a past event. This may lead to intrusive sensory-perceptual information being experienced as an actual voice in the present moment.

While the present methodology cannot demonstrate aetiological causality, the high rate of phenomenological links between trauma and voices does provide some support for trauma-informed cognitive behavioural models in the treatment of hallucinations (Freeman, Garety, Kuipers, Fowler, & Bebbington, 2002; Garety et al., 2001; Hardy, 2017; Morrison, 2001; Morrison et al., 2003; Waters, Badcock, Michie, & Maybery, 2006) that postulate that abovementioned trauma-related psychological mechanisms may give rise to hallucinations as well as to their content and responses to them. These trauma-related psychological mechanisms may be identified and targeted using collaborative psychological formulation (Hardy et al., 2019; Van Den Berg, van de Giessen, & Hardy, 2019), which include trauma-related victimisation, physiological-behavioural response, emotional response, and cognitive response themes, and links with these themes experienced in voices. Established psychological therapies already recognise how trauma shapes voice hearing (e.g. cognitive behavioural therapy for psychosis; Turner, Burger, Smit, Valmaggia, and van der Gaag, 2020) with therapeutic innovations increasingly focused on targeting the autobiographical context of voice hearing (Brand, Bendall, Hardy, Rossell, & Thomas, 2021; Craig et al., 2018; Paulik, Steel, & Arntz, 2019; Varese et al., 2021; Ward et al., 2020). Our recent research also supports that many people have trauma-explanations for their voices, which suggests they are likely willing to engage in trauma-informed or trauma-focused therapy (Tolmeijer et al., 2021).

Besides the strengths of the study, some limitations have to be considered. This study investigated phenomenological trauma–voice links within a group of voice-hearers with a history of interpersonal trauma and frequent and distressing voices thereby excluding voice-hearers with other types of trauma and with less frequent or non-distressing voices. Rates of phenomenological links presented here may therefore not be representable for the entire group of voice-hearers, although similar rates of childhood trauma have been reported in clinical and non-clinical voice-hearing groups (Daalman & Diederen, 2013). While trauma and voices were rigorously investigated, the focus was on participants' most distressing trauma and voices. The selection criterion of having experienced the most distressing interpersonal traumatic event before the onset of voices also excluded people with traumatic experiences later in life. However, traumatic experiences after the onset of voices may also shape the content and related consequences of voices through mirroring interactions during these events. Therefore, results may actually underreport phenomenological links between trauma and voices. Since the present sample included people with distressing and frequent voices and severe and repeated traumatic experiences, and researchers were only provided with brief descriptions of these experiences, many independently rated themes were either highly prevalent or absent such that OR estimates could only be calculated for some themes. Additionally, findings reported at the 0.05 significance level should also be seen as explorative and suggestive for hypotheses in future research. Future studies should therefore replicate the results in people with less distressing and frequent voices and voice-hearers in the general population. Future studies can apply the present methodology to further investigate the phenomenological links between multiple traumas and voices.

In summary, many voice hearers have experienced trauma and both voice-hearers and researchers commonly identify phenomenological links between trauma and voices. The findings suggest that the phenomenology of trauma and voices is commonly characterised by cognitive-affective psychological threat and provide insight into how trauma-related meaning and affect can become embodied in the relational experience with voices. The findings highlight the importance of collaboratively developing psychological case formulations of voices to understand phenomenological links between trauma and voices, which can be addressed in therapy. Trauma-related survival strategies and negative self-beliefs might be valuable therapeutic targets. The findings support that voice-hearer's perspectives should be central in the content and course of psychological interventions, especially where voices may be linked to traumatic experiences and their impact. Additionally, the study provides a framework for investigating and reporting thematic, relational, content, and identity links, which may be refined in future research, for example by assessing multiple traumatic events and voices and evaluating its psychometric properties further to this initial development study. Our theory-driven coding frame could also be adapted in the future to form a questionnaire for clinically understanding trauma–voice links. It is our hope that our method promotes further research on the phenomenology of trauma and hallucinations and helps refine trauma-informed psychological interventions offered to people with distressing voices.

## Supplementary material

For supplementary material accompanying this paper visit https://doi.org/10.1017/S0033291721004955. click here to view supplementary material
