# Supplementary material for: Voice phenomenology as a mirror of the past
Source: Psychol Med. 2022 Jan 7;53(7):2954–62. doi: 10.1017/S0033291721004955 (PMC10235665; doi:10.1017/S0033291721004955)
Supplement: Supplementary file 1 [file S0033291721004955sup001.docx]

**Supplementary Materials**

**The theory-driven structured coding frame**

**Section I**

*Can you provide a short, detailed description of your most distressing voices?*

*It has to contain the following elements:*

*1. Identity*

*2. Type of voice (offensive, commanding, commenting)*

*3. Content of the voices (e.g., what do they comment on, what kind of insults are they making, what kind of orders do they give)*

*4. Meaning, interpretations, thoughts*

*5. Emotional response (scared, angry, happy, sad, ashamed)*

*6. Physical response (not always present)*

*7. Behavioural response (both active and passive)*

*Note: write it down in this order, with each section having its own sentence. Write this down in the first person.*

*Write down the answer in a maximum of 100 words*

|  |
| --- |

*Please answer the following questions in relation to your most distressing voices:*

| **1. How do the voices treat you?** | No, not at all Yes, very much | | | | | | |
| --- | --- | --- | --- | --- | --- | --- | --- |
| a. They do things to me or pressure me to do things I don’t want to do. | 0 | 1 | 2 | 3 | 4 | | 5 |
| b. They are violent, physically threaten or hurt me. | 0 | 1 | 2 | 3 | 4 | | 5 |
| c. They criticise me, insult me, put me down, or blame me for things that are not my fault. | 0 | 1 | 2 | 3 | 4 | | 5 |
| d. They humiliate, embarrass or shame me in front of others. | 0 | 1 | 2 | 3 | 4 | | 5 |
| **2. How do you react to the voices and how does your body respond to the voices?** | No, not at all Yes, very much | | | | | | |
| a. I do what I am told or what is expected of me, or just let it happen. | 0 | 1 | 2 | 3 | | 4 | 5 |
| b. I try to fight back and stop it happening. | 0 | 1 | 2 | 3 | | 4 | 5 |
| c. I try to avoid it, such as by escaping, running away, or being somewhere safer. | 0 | 1 | 2 | 3 | | 4 | 5 |
| d. I shut off, go numb, blank out, feel detached from or not part of me, or my body feels paralysed. | 0 | 1 | 2 | 3 | | 4 | 5 |
| e. I try to ignore what is happening to not let it affect me. | 0 | 1 | 2 | 3 | | 4 | 5 |
| **3. How do you feel when you hear the voices?** | No, not at all Yes, very much | | | | | | |
| a. Afraid, fearful, scared, or stressed. | 0 | 1 | 2 | 3 | | 4 | 5 |
| b. Sad, low, down or depressed. | 0 | 1 | 2 | 3 | | 4 | 5 |
| c. Angry, irritable or frustrated. | 0 | 1 | 2 | 3 | | 4 | 5 |
| d. Ashamed. | 0 | 1 | 2 | 3 | | 4 | 5 |
| e. Guilty. | 0 | 1 | 2 | 3 | | 4 | 5 |
| **4. What do you think when you hear the voices?** | No, not at all Yes, very much | | | | | | |
| a. I am powerless. | 0 | 1 | 2 | 3 | | 4 | 5 |
| b. I am in danger, others are out to get me or want to cause me harm. | 0 | 1 | 2 | 3 | | 4 | 5 |
| c. I am bad, worthless, wrong, stupid, or dirty. | 0 | 1 | 2 | 3 | | 4 | 5 |
| d. Others will reject me or judge me negatively. | 0 | 1 | 2 | 3 | | 4 | 5 |

*Can you provide a short, detailed description of your worst traumatic experience or category of experiences (e.g., repeated experience of the same sort)? Which person or group of people treated you the worst during these events?*

*It has to contain the following elements:*

*1. Identity perpetrator*

*2. Type of trauma (general)*

*3. Content of the trauma (what happened during the traumatic event(s))*

*4. Meaning, interpretations, thoughts at the time*

*5. Emotional response (scared, angry, happy, sad, ashamed)*

*6. Physical response (not always present)*

*7. Behavioural response (both active and passive)*

*Note: write it down in this order, with each section having its own sentence. Write this down in the first person.*

*Write down the answer in a maximum of 100 words*

|  |
| --- |

*The following questions are about the experiences you described previously, concerning difficult or traumatic events that involved other people. Please answer each question in relation to your worst experiences:*

| **1. How did people treat you during these events?** | No, not at all Yes, very much | | | | | |
| --- | --- | --- | --- | --- | --- | --- |
| a. They did things to me or pressured me to do things I did not want to do (for instance sexually). | 0 | 1 | 2 | 3 | 4 | 5 |
| b. They were violent, physically threatened or hurt me. | 0 | 1 | 2 | 3 | 4 | 5 |
| c. They criticised me, insulted me, put me down, or blamed me for things that were not my fault. | 0 | 1 | 2 | 3 | 4 | 5 |
| d. They humiliated, embarrassed or shamed me in front of others. | 0 | 1 | 2 | 3 | 4 | 5 |
| **2. How did you react and how did your body respond during these events?** | No, not at all Yes, very much | | | | | |
| a. I did what I was told or what was expected of me, or just let it happen. | 0 | 1 | 2 | 3 | 4 | 5 |
| b. I tried to fight back and stop it happening. | 0 | 1 | 2 | 3 | 4 | 5 |
| c. I tried to avoid it, such as by escaping, running away, or being somewhere safer. | 0 | 1 | 2 | 3 | 4 | 5 |
| d. I shut off, went numb, blanked out, felt detached from or not part of me, or my body felt paralysed. | 0 | 1 | 2 | 3 | 4 | 5 |
| e. I tried to ignore what was happening to not let it affect me. | 0 | 1 | 2 | 3 | 4 | 5 |
| **3. What did you feel during these events?** | No, not at all Yes, very much | | | | | |
| a. Afraid, fearful, scared, or stressed. | 0 | 1 | 2 | 3 | 4 | 5 |
| b. Sad, low, down or depressed. | 0 | 1 | 2 | 3 | 4 | 5 |
| c. Angry, irritable or frustrated. | 0 | 1 | 2 | 3 | 4 | 5 |
| d. Ashamed. | 0 | 1 | 2 | 3 | 4 | 5 |
| e. Guilty. | 0 | 1 | 2 | 3 | 4 | 5 |
| **4. What did you think during or after these events?** | No, not at all Yes, very much | | | | | |
| a. I was powerless/ | 0 | 1 | 2 | 3 | 4 | 5 |
| b. I was in danger, others were out to get me or wanted to cause me harm. | 0 | 1 | 2 | 3 | 4 | 5 |
| c. I was bad, worthless, wrong, stupid, or dirty. | 0 | 1 | 2 | 3 | 4 | 5 |
| d. I was afraid that others would reject me or judge me negatively. | 0 | 1 | 2 | 3 | 4 | 5 |

**Section II**

*Please answer the following questions about your most distressing voices and worst experiences of events involving others:*

|  | No, not at all Yes, very much | | | | | |
| --- | --- | --- | --- | --- | --- | --- |
| a. Do the voices sound like or belong to people involved in any of the events? | 0 | 1 | 2 | 3 | 4 | 5 |
| b. Do the voices every say exactly the same things that were said to you during the events? | 0 | 1 | 2 | 3 | 4 | 5 |
| c. Is the way the voices treat you or the way that they behave similar to the way people treated you during any of the events? | 0 | 1 | 2 | 3 | 4 | 5 |
| d. Do you react to or cope with the voices in a similar way to how you reacted or coped during any of the events? | 0 | 1 | 2 | 3 | 4 | 5 |
| e. Do the voices make you feel the same as how you felt during the events? | 0 | 1 | 2 | 3 | 4 | 5 |

**The theory-driven structured coding frame adapted for researchers**

**Section I: independent links**

*Description of the most distressing voice(s) (maximum of 100 words)*

|  |
| --- |

*The following questions are about the description of the most distressing voices:*

| **1. Victimisation type** | **No** | **Yes** |
| --- | --- | --- |
| a. Coercion: coercion including being involuntarily confined. |  |  |
| b. Violence: actual or threatened violence. |  |  |
| c. Criticism/Blame: criticism or blame, including negative comments. |  |  |
| d. Humiliation: being socially devaluated in the presence of others. |  |  |
| **2. Physiological-behavioural response** |  |  |
| a. Submission: passive response. |  |  |
| b. Fight: fighting, resisting, or attempting to control the situation. Thinking out loud that the voices were wrong was also considered to be resistance if clearly directed to the voices. |  |  |
| c. Flight: running/fleeing/avoiding/negative experiential avoidance. |  |  |
| d. Freeze: emotional detachment, feeling numb, freezing, depersonalization, derealisation, fainting or tonic immobility. |  |  |
| e. Ignoring: coping by ignoring. |  |  |
| **3. Emotional response** |  |  |
| a. Anxiety: clear reference to the emotion anxiety. |  |  |
| b. Sadness: clear reference to the emotion sadness. |  |  |
| c. Anger: clear reference to the emotion anger. |  |  |
| d. Shame: clear reference to the emotion shame. |  |  |
| e. Guilt: clear reference to the emotion guilt. |  |  |
| **4. Cognitive response** |  |  |
| a. Powerless: clear reference to the cognition powerless, or the emotion and/or behaviour strongly implies an associated cognitive response. |  |  |
| b. Threatened: clear reference to the cognition threatened, or the emotion and/or behaviour strongly implies an associated cognitive response. |  |  |
| c. Negative self: clear reference to the cognition negative-self, or the emotion and/or behaviour strongly implies an associated cognitive response. |  |  |
| d. Fear of rejection: clear reference to the cognition fear of rejection, or the emotion and/or behaviour strongly implies an associated cognitive response. |  |  |

*Description of the most difficult or traumatic experiences involving other people (maximum of 100 words)*

|  |
| --- |

*The following questions are about the description of the most difficult or traumatic events that involved other people.*

| **1. Victimisation type** | **No** | **Yes** |
| --- | --- | --- |
| a. Coercion: coercion including being involuntarily confined. |  |  |
| b. Violence: actual or threatened violence. |  |  |
| c. Criticism/Blame: criticism or blame, including negative comments. |  |  |
| d. Humiliation: being socially devaluated in the presence of others. |  |  |
| **2. Physiological-behavioural response** |  |  |
| a. Submission: passive response. |  |  |
| b. Fight: fighting, resisting, or attempting to control the situation. |  |  |
| c. Flight: running/fleeing/avoiding/negative experiential avoidance. |  |  |
| d. Freeze: emotional detachment, feeling numb, freezing, depersonalization, derealisation, fainting or tonic immobility. |  |  |
| e. Ignoring: coping by ignoring. |  |  |
| **3. Emotional response** |  |  |
| a. Anxiety: clear reference to the emotion anxiety. |  |  |
| b. Sadness: clear reference to the emotion sadness. |  |  |
| c. Anger: clear reference to the emotion anger. |  |  |
| d. Shame: clear reference to the emotion shame. |  |  |
| e. Guilt: clear reference to the emotion guilt. |  |  |
| **4. Cognitive response** |  |  |
| a. Powerless: clear reference to the cognition powerless, or the emotion and/or behaviour strongly implies an associated cognitive response. |  |  |
| b. Threatened: clear reference to the cognition threatened, or the emotion and/or behaviour strongly implies an associated cognitive response. |  |  |
| c. Negative self: clear reference to the cognition negative-self, or the emotion and/or behaviour strongly implies an associated cognitive response. |  |  |
| d. Fear of rejection: clear reference to the cognition fear of rejection, or the emotion and/or behaviour strongly implies an associated cognitive response. |  |  |

**Section II: dependent links**

*The following questions are about the most distressing voices and worst experiences of events involving others.*

|  | **No** | **Yes** |
| --- | --- | --- |
| a. Relational link: similar interaction with or response to voice(s). |  |  |
| b. Content link: voice content is exactly the same as the trauma content. |  |  |
| c. Identity link: voice identity is the same as the perpetrator identity. |  |  |
